# Selectivity Tuning by Natural Deep Eutectic Solvents (NADESs) for Extraction of Bioactive Compounds from *Cytinus hypocistis*—Studies of Antioxidative, Enzyme-Inhibitive Properties and LC-MS Profiles

**DOI:** 10.3390/molecules27185788

**Published:** 2022-09-07

**Authors:** Gokhan Zengin, María de la Luz Cádiz-Gurrea, Álvaro Fernández-Ochoa, Francisco Javier Leyva-Jiménez, Antonio Segura Carretero, Malwina Momotko, Evren Yildiztugay, Refik Karatas, Sharmeen Jugreet, Mohamad Fawzi Mahomoodally, Grzegorz Boczkaj

**Affiliations:** 1 Department of Biology, Science Faculty, Selcuk University, Konya 42130, Turkey; 2 Department of Analytical Chemistry, Faculty of Sciences, University of Granada, Fuentenueva s/n, 18071 Granada, Spain; 3 Department of Analytical Chemistry and Food Science and Technology, University of Castilla-La Mancha, Ronda de Calatrava 7, 13071 Ciudad Real, Spain; 4 Regional Institute for Applied Scientific Research (IRICA), Area of Food Science, University of Castilla-La Mancha, Avenida Camilo Jose Cela, 10, 13071 Ciudad Real, Spain; 5 Department of Process Engineering and Chemical Technology, Faculty of Chemistry, Gdansk University of Technology, G. Narutowicza St. 11/12, 80-0233 Gdansk, Poland; 6 Department of Biotechnology, Science Faculty, Selcuk University, Konya 42130, Turkey; 7 Department of Health Sciences, Faculty of Medicine and Health Sciences, University of Mauritius, Réduit 80837, Mauritius; 8 Center for Transdisciplinary Research, Department of Pharmacology, Saveetha Institute of Medical and Technical Science, Saveetha Dental College, Chennai 600077, India; 9 Centre of Excellence for Pharmaceutical Sciences (Pharmacen), North West University, Potchefstroom 2520, South Africa; 10 Department of Sanitary Engineering, Faculty of Civil and Environmental Engineering, Gdansk University of Technology, G. Narutowicza St. 11/12, 80-0233 Gdansk, Poland; 11 Advanced Materials Center, Gdansk University of Technology, G. Narutowicza St. 11/12, 80-233 Gdansk, Poland

**Keywords:** NADES, total polyphenolic content, antioxidants, enzyme inhibition, functional food, natural medicine, Alzheimer cholinesterase inhibitors

## Abstract

In the present study, the extracts of *Cytinus hypocistis* (L.) L using both traditional solvents (hexane, ethyl acetate, dichloromethane, ethanol, ethanol/water, and water) and natural deep eutectic solvents (NADESs) were investigated in terms of their total polyphenolic contents and antioxidant and enzyme-inhibitive properties. The extracts were found to possess total phenolic and total flavonoid contents in the ranges of 26.47–186.13 mg GAE/g and 0.68–12.55 mg RE/g, respectively. Higher total phenolic contents were obtained for NADES extracts. Compositional differences were reported in relation to antioxidant potential studied by several assays (DPPH: 70.19–939.35 mg TE/g, ABTS: 172.56–4026.50 mg TE/g; CUPRAC: 97.41–1730.38 mg TE/g, FRAP: 84.11–1534.85 mg TE/g). Application of NADESs (choline chloride—urea 1:2, a so-called Reline) allowed one to obtain the highest number of extracts having antioxidant potential in the radical scavenging and reducing assays. NADES-B (protonated by HCl L-proline-xylitol 5:1) was the only extractant from the studied solvents that isolated a specific fraction without chelating activity. Reline extract exhibited the highest acetylcholinesterase inhibition compared to NADES-B and NADES-C (protonated by H_2_SO_4_ L-proline-xylitol 5:1) extracts, which showed no inhibition. The NADES extracts were observed to have higher tyrosinase inhibitory properties compared to extracts obtained by traditional organic solvents. Furthermore, the NADES extracts were relatively better inhibitors of the diabetic enzymes. These findings provided an interesting comparison in terms of total polyphenolic content yields, antioxidant and enzyme inhibitory properties (cholinesterase, amylase, glucosidase, and tyrosinase) between traditional solvent extracts and NADES extracts, used as an alternative. While the organic solvents showed better antioxidant activity, the NADES extracts were found to have some other improved properties, such as higher total phenolic content and enzyme-inhibiting properties, suggesting functional prospects for their use in phytonutrient extraction and fractionation. The obtained results could also be used to give a broad overview of the different biological potentials of *C. hypocistis*.

## 1. Introduction

The genus *Cytinus*, composed of endophytic parasitic plants (family: *Cytinaceae*), bears eight recognised species distributed around two centres of diversity: one in southern Africa and Madagascar, and one in the Mediterranean region [1].

Indeed, folkloric medicine has dedicated substantial consideration to this genus. These plants have been used traditionally for treating dysentery, including their ability to soothe inflammations of the eyes and throat. Some ethnobotanical reviews have also noted the use of *Cytinus* juice as an astringent, a haemostatic, and a tonic substance. They are also used as a scar-healing agent, whereby the scalp pulp is applied daily on corns and calluses, skin and swollen mucous membranes as an astringent and anti-inflammatory therapy [1,2].

Some studies have also pointed out these plants’ beneficial potential and suggested their antimicrobial effects over a range of bacterial strains and antioxidant activities [2,3,4]. Furthermore, they have been highlighted as good sources of biologically active ingredients of cosmeceutical interest [2,3,5]. In fact, their biological activities have been correlated with their high tannin content. For instance, hydrolysable tannins were found to be the active cytotoxic compounds identified in three *Cytinus* taxa and were assessed against a wide variety of cancer cell lines [6]. In another study, the tested extract of *C.* *hypocistis* was found to exhibit anti-inflammatory activity and effective cytotoxicity againcst tumour cells, while it showed the lowest cytotoxicity on a non-tumour cell line, and interestingly, hydrolysable tannins and flavonoids were also identified as the main groups in the extract [7].

Plants possess a diverse range of such bioactive constituents. However, their availability strongly depends on the extraction techniques used, among other factors. Even though to date numerous methods have been developed and upgraded, there is still a need to achieve a standardised solution with high consideration for the extraction of bioactive compounds from plants [8].

In addition, there is growing pressure to investigate alternative solvents that retain the technological advantages of organic solvents while posing less risk to human health and the environment. Deep eutectic solvents (DESs) and their specialised form obtained from compounds of natural origin—natural DESs (NADESs)—have shown the most promise in the field of green chemistry because they are abundant, inexpensive, recyclable, and appealing for a wide range of applications (food, cosmetic, and pharmaceutical). DESs have already proved to have several advantages in separation science, especially in terms of unusual selectivity useful in chromatography [9,10] and extraction [11,12], as well as membrane processes [13,14]. Many studies have effectively employed NADES extraction to gain high-quality extracts from numerous plants, including medicinal plants [15,16,17]. NADESs’ potential biological activity, bioavailability, and the availability of a variety of solvent combinations for its preparation are also intriguing characteristics. Therefore, NADES extraction is a cutting-edge technique that has piqued the interest of researchers and already exhibited great promise in the extraction and isolation of bioactive compounds from plants [17]. 

Therefore, the aim of the present study is to compare characteristics of extracts obtained by means of NADESs with classic organic solvents. For this purpose, the *Cytinus* *hypocistis* (L.) L. extracts were analysed in respect to their LC-MS profiles, the total phenolic (TPC) and flavonoid (TFC) contents, and antioxidant as well as enzyme-inhibitive properties.

## 2. Results and Discussion

### 2.1. Phytochemical Profiles

Natural products are important sources for drug development. Thus, it is of crucial importance to develop effective methods to extract and isolate these bioactive products. Indeed, the lab-intensive and laborious extraction and isolation processes have been a major challenge in the application of natural products in drug development. There is an urgent need to develop efficient and selective methods for this [18]. In this respect, different types of solvents have been widely used for the extraction of phytochemicals, whereby dried plant powders are used to extract bioactive phytochemicals and remove the Interference of water concomitantly. The solvents used for the extraction of biomolecules from plants are chosen based on the polarity of the desired solute. For example, a solvent with the same polarity as the solute will effectively dissolve the solute. Several solvents can be used sequentially to limit the number of analogous compounds in the desired yield [19]. 

Solubility, bioavailability, and stability are all factors in the pharmacological efficacy of plant extracts and their bioactive principles. Natural deep eutectic solvents (NADESs) are considered as green solvents to enhance the extraction performance of plant metabolites [16]. As functional liquid media, NADESs can dissolve both natural and synthetic substances with low water solubility. Hence, they are alternative candidates for applications with some organic solvents, as well as ionic liquids [20], indicating the enormous potential for NADESs to be utilised in the development of pharmaceutical formulations, such as nutraceuticals derived from plant-based metabolites [16]. Thus, in this study, both traditional solvents and NADESs were used to prepare *C.* *hypocistis* extracts and to compare their overall performance in terms of their bioactive content yields and biological activities.

**Figure 1:**
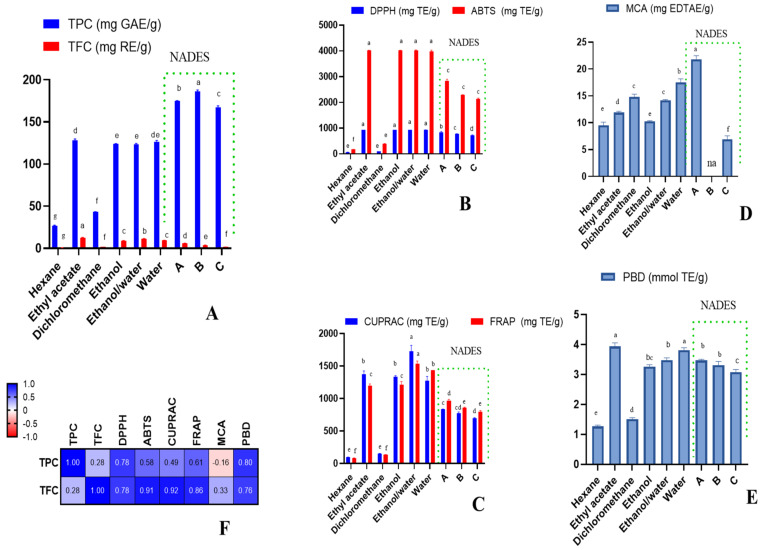
Total phenolic and flavonoid content (**A**), radical scavenging ability (**B**), reducing power (**C**), metal-chelating ability (MCA) (**D**), total antioxidant ability (by phosphomolybdenum assay (PBD)) (**E**), and Pearson’s correlations between total bioactive compounds and antioxidant assays (*p* < 0.05) (**F**). na: not active; different letters in column for same assays indicate significant differences in the extracts (*p* < 0.05).

In the phytochemical studies, the investigation of the polyphenol content present in plant extracts is an important part of assessing their biological properties. In this study, the extracts were found to possess TPC and TFC in the range of 26.47–186.13 mg GAE/g and 0.68–12.55 mg RE/g, respectively. Interestingly, the extracts obtained by NADESs yielded higher TPC (167.57–186.13 mg GAE/g), followed by ethyl acetate, water, ethanol, and ethanol/water extracts (123.51–127.83 mg GAE/g). On the other hand, the dichloromethane and hexane extracts yielded low TPC. In the TFC assay, the highest yield was obtained by ethyl acetate and ethanol/water extracts, followed by water and ethanol extracts, while the least TFC was yielded by hexane, NADES-C, and dichloromethane extracts (Figure 1, Table S1). The variation in total polyphenol content clearly varied with the polarity of the solvents used. However, not only the polarity of the solvents, but also other parameters such as pH, extraction time, methods, and temperature can affect the extraction yield and total phenolic content [21,22,23].

### 2.2. Characterization of Polar Bioactive Compounds from C. hypocistis Extracts by UPLC-ESI-QTOF-MS

**Figure 2:**
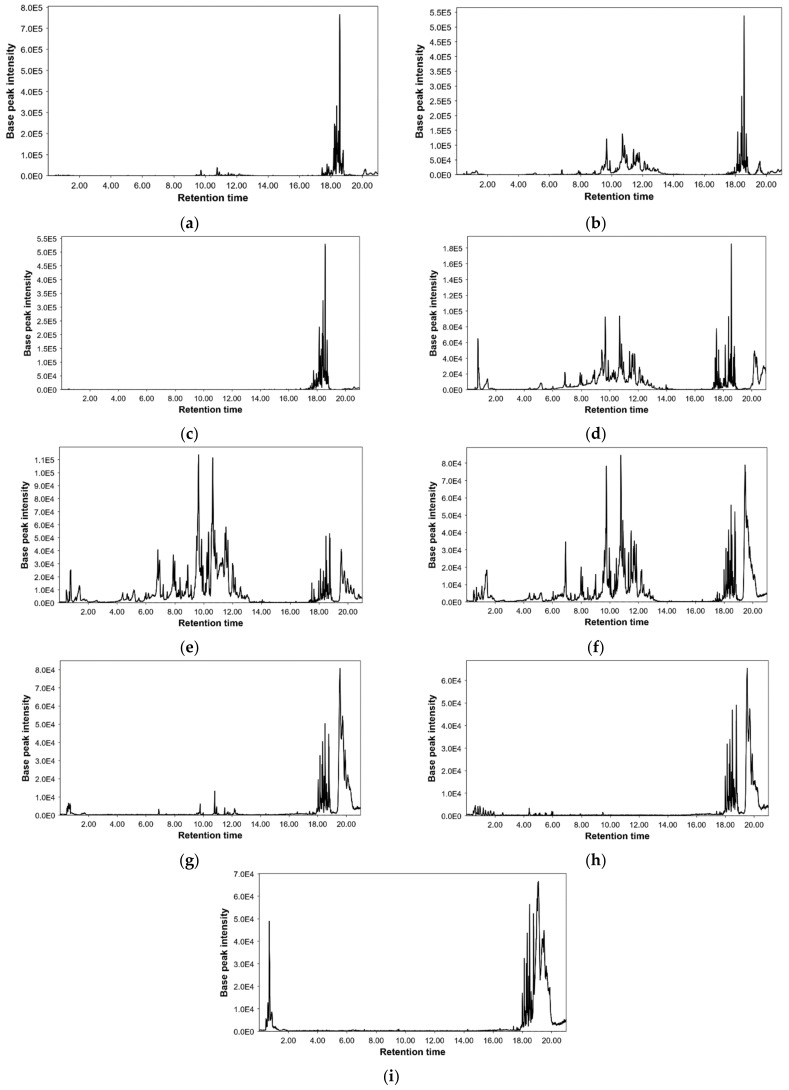
Base peak chromatograms from Cytinus (**a**) hexane, (**b**) ethyl acetate, (**c**) dichloromethane, (**d**) ethanol, (**e**) ethanol/water, (**f**) water, (**g**) NADES-A, (**h**) NADES-B, and (**i**) NADES-C extracts by UPLC-MS.

Following the described LC-MS method, all extracts were analysed, resulting in a total of 148 detected compounds. Figure 2 shows the base peak chromatograms performed for each extraction condition and Table S2 summarises all information about detected compounds such as retention time, *m*/*z* ratio, error in ppm, molecular formula, and name of each proposed compound. In addition, peak numbers were assigned according to their elution order. The peak areas of the detected compounds are given in Table S3.

It is worth to note that to our knowledge, there is little reference to a comprehensive characterisation of *C. hypocistis* extracts [1]. For this reason, our work is especially relevant. Considering the accurate mass spectra information and data previously reported by literature, 136 compounds were tentatively annotated in this study. Only one common molecular feature was detected among all the different extractions; however, this molecular feature could not be annotated and remained as an unknown compound (Table 1). This could be explained mainly by the differences in the extraction efficiencies of the different solvents used, also considering the different physicochemical properties of the compounds present in the matrix.

Overall, the tentative characterisation allowed one to classify the compounds in five major groups: gallotannins, ellagitannins, flavonoids, fatty acids, and other compounds, with it being important to note that gallotannins were the most important one, with 61 compounds included. These were mainly annotated as mono-, di-, tri-, tetra-, penta-, hexa-, and hepta-galloyl hexoside. In addition, different isomers were also annotated for each of these types of chemical structures. Silva et al., reported in 2020 from mono- to penta-galloyl hexosides in different parts (petals, stalks, and nectar) of *C. hypocistis* [24]. However, all these compounds were previously reported in other sources such as *Magnifera indica* L. kernels and peels [25,26], *Rhodiola crenulate* roots [27], *Rhodiola rosea* roots, leaves, stems and flowers [27], Paeonia plants [28], and *Pistacia vera* leaves [29], among others [30]. Table 1 shows that a major number of these gallotannins were found in ethyl acetate, ethanol, water/ethanol, and water extracts. In recent years, several authors have reported a wide range of biological properties of these galloyl hexoside derivatives [27,29,31,32]. Among other gallic acid derivatives presented in the gallotannins group, three isomers from neochebulagic acid corresponding to peaks 60, 61, and 70 have been also annotated. These compounds were reported in *Terminalia chebula* Retz. Playing a role in the intestinal glucose transport [33]. Moreover, several compounds previously found in *Trapa quadrispinosa* pericarps were detected in our extracts, such as peaks 49, 67, 75, and 87 (digalloyl-lactonised valoneoyl-d-glucose isomers); peaks 71, 92, and 98 (trigalloyl-lactonised valoneoyl glucose isomers); and peaks 78, 85, and 102 (galloyl-penta-hydroxy-benzoic-brevifolincarboxyl-glucose isomers) [34].

As the second most important group, ellagitannins contains 30 compounds, which were annotated as different mono-, di-, and tri-galloyl-DHHDP-glucose isomers and digalloyl-HHDP-iso DHDG-glucose isomers. Besides these compounds, other ellagitannins such as terflavin B, phyllanthussin C, geraniin, and balanophlorotannin E isomers have been previously reported in various *Terminalia* species and in *Trapa* species [34,35]. In case of our species, only peaks corresponding to *m*/*z* 937 and 783 were annotated [24].

Regarding flavonoids, catechin and epicatechin as flavan-3-ols were found; quercetin and two isorhamnetin glucoside isomers as flavonols were also detected, mostly with water, ethanol, their mixture, and ethylacetate.

On the other hand, hexane and dichloromethane extracts presented the major number of fatty acids (Peaks 118, 119, 121–132, 135–141, 144–148). Regarding NADESs, the three extracts showed the lower number of features, and many of the features obtained could not be annotated such as peaks 7 and 9 (unknowns 3 and 4) were only found in NADES-C extract. The same happened for peak 2 (unknown 2); this compound was only detected in NADES-B. Among them, NADES-A presented high number of features corresponding mainly to gallotannins. This highlights the potential of NADESs to obtain extracts containing additional bioactive compounds and to “tailor” the properties of extracts. Secondly, extraction by two NADESs sequentially should allow for the fractionation of bioactive compounds.

Other compounds have been also annotated, although they have not been classified in specific groups due to the low number and the high range of structures. For instance, organic acids (quinic and fukiic acids); simple phenols (gallic and ellagic acids); and two alkyl-phenylketones, namely brevifolin and brevifolin carboxylic acid [36], were included in this group. In addition, three isomers from galloylnorbergenin, antioxidant isocoumarins that were previously found in leaves of *Diospyros gilletii* De Wild [37] were also included.

### 2.3. Antioxidant Effects

Antioxidants are important chemical substances that occur naturally in food and can reduce or prevent oxidative stress of the physiological system as the body continuously produces free radicals. Oxidative stress plays a key role in the development of chronic and degenerative diseases such as cancer, autoimmune diseases, and neurodegenerative and cardiovascular diseases. The human body has a variety of mechanisms to counter oxidative stress by producing antioxidants that are either naturally produced in situ or supplied externally through foods such as plants, as a rich source of naturally produced antioxidants. Hence, antioxidants acting as free radical scavengers can eventually help to avert and repair cellular damage generated by these radicals [38,39].

Herein, moderate-to-very-potent antioxidant activity was noted for *C. hypocistis* extracts. For instance, in the radical scavenging assays, the ethanol/water, ethanol, ethyl acetate, water, and NADES-A extracts demonstrated very high antioxidant potential (DPPH: 829.11–939.35 mg TE/g; ABTS: 2830.66–4026.50 mg TE/g), followed by NADES-C and NADES-B (DPPH: 701.49 and 767.55 mg TE/g; ABTS: 2134.94 and 2285.15 mg TE/g, respectively). On the other hand, a much lower scavenging ability was displayed by the hexane and dichloromethane extracts (DPPH: 70.19 and 93.25 mg TE/g; 172.56 and 398.03 mg TE/g, respectively), compared to the other extracts. The same trend was observed with the reducing assays, whereby the ethanol/water, ethanol, ethyl acetate, water, and NADES-A extracts showed significant reducing capacity in the range of 1730.38–1377.38 mg TE/g and 968.98–1534.85 mg TE/g in CUPRAC and FRAP assays, respectively, whereas a lower reducing activity was noted for NADES-B and NADES-C and a much lower content for hexane and dichloromethane extracts (CUPRAC: 97.41–774.94 mg TE/g; FRAP: 84.11–860.90 mg TE/g) (Figure 1, Table S4). It is clear that most valuable bioactive components are extracted by relatively polar extracts, while low-polarity solvents such as *n*-hexane and dichloromethane are not effective in this case. In Figure 1, Pearson’s correlation analysis indicates a linear correlation (R > 0.7) between total flavanoid content and radical scavenging and reducing power assays.

While only NADES-B did not possess metal-chelating activity, all the other extracts showed metal-chelating potential in the range of 6.87–21.76 mg EDTAE/g. The extracts also demonstrated total antioxidant capacity in phosphomolybdenum assay (1.27–3.94 mmol TE/g). The lowest total antioxidant capacity was revealed for hexane and dichloromethane extracts (Figure 1). This part of the research reveals a possibility to isolate metal-chelating components by step extraction using NADES-B at the first stage, followed by a second extractant that is effective to extract this specific group of compounds present in the studied plant.

### 2.4. Enzyme Inhibitory Effects

Low levels of the neurotransmitter acetylcholine, oxidative stress, and inflammation in the central nervous system (CNS) are hallmarks of Alzheimer’s disease (AD), a progressive neurodegenerative disease. To date, patients diagnosed with AD are only offered enzyme inhibitors (acetylcholinesterase/butyrylcholinesterase, or AChE/BChE) for treatment [40]. Hence, as mechanism of pharmacological action, these cholinesterase inhibitors are able to modify cholinergic signalling by disrupting the degradation of acetylcholine [41].

**Figure 3:**
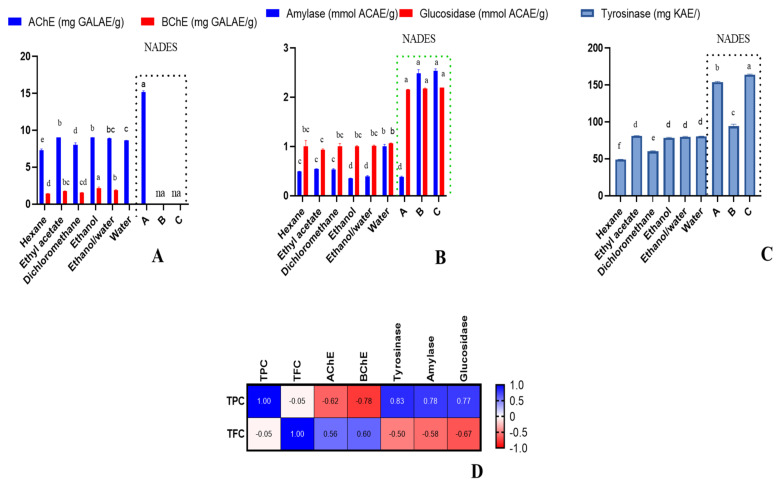
Cholinesterase inhibitory effects (**A**), amylase and glucosidase inhibitory effects (**B**), tyrosinase inhibitory effects (**C**), Pearson’s correlations between total bioactive compounds and enzyme inhibitory assays (*p* < 0.05) (**D**). na: not active. Different letters in column for same assays indicate significant differences in the extracts (*p* < 0.05).

All extracts, except NADES-B and NADES-C extracts, possessed anti-AChE activity (7.32–15.16 mg GALAE/g). Interestingly, the highest anti-AChE activity was revealed for NADES-A. It is particularly important observation, as components of NADES-A—choline chloride and urea—are nontoxic; thus, such obtained extracts could be further used without removal of NADES. On the other hand, only the extracts prepared in the traditional way, with solvents hexane, ethyl acetate, dichloromethane, ethanol, and ethanol/water, displayed anti-BChE potential (1.39–2.13 mg GALAE/g). The water extract and the NADES extracts showed no anti-BChE activity. It follows from the ionic nature of the used NADESs, which in this case was not favourable for extraction. The ethanol extract demonstrated relatively higher BChE inhibitory effect compared to the other extracts (Figure 3).

Tyrosinase is the rate-limiting enzyme in melanin synthesis. Melanin is synthesised in human melanocytes when tyrosine is hydroxylated to l-DOPA, which is then oxidised to dopaquinone and polymerises to form melanin. Melasma, melanoma, and freckles are just some of the dermatological conditions that can develop when melanin production increases too rapidly. However, tyrosinase in plant-based foods oxidises phenolic compounds into quinones. The former reacts with amino acids and proteins to produce brown/black pigments, a process known as enzymatic browning, which is one of the most pressing problems in the food industry and the source of 50 percent of the industry’s economic losses. In addition, browning reduces the food’s nutritional value and safety because it leads to the loss of vitamin C, antioxidants, and other nutrients, and can even lead to the production of antinutritional and toxic substances. Consequently, tyrosinase inhibition is seen as an efficient method for preventing hyperpigmentation in the pharmaceutical industry and delaying enzymatic browning, which is helpful in the food industry [42].

In the current investigation, all the studied extracts were found to possess antityrosinase activity (49.14–153.97 mg KAE/g). However, NADES-A exhibited the highest inhibitory activity against tyrosinase, while the hexane extract displayed the lowest (Figure 3). On this basis, it is clear that polar NADES-A, as well as other polar solvents, should be preferred for the extraction of bioactive compounds responsible for tyrosinase activity.

Interestingly, in the study by Zucca et al. [2], the ethanolic extract of *C. hypocistis* showed the highest tyrosinase inhibition activity, compared to cyclohexane and water extracts, probably due to being predominantly rich in polyphenols, in most part hydrolysable tannins. *C. hypocistis* aerial part extract was also reported to show tyrosinase inhibition of 80%, when tested at 50 μg/mL [43]. Furthermore, a linear correlation was obtained between enzymatic activities and increasing TPC and TFC. The reason could be a specific class of polyphenols acting against tyrosinase through a competitive inhibition mechanism, thus interfering with the biological function of tyrosinase, which is a polyphenoloxidase [43].

The inhibition of the carbohydrate-digesting enzymes alpha-glucosidase and alpha-amylase is an important strategy for controlling blood glucose levels in patients with Type 2 diabetes and borderline diabetes, because it significantly reduces the postprandial rise in blood glucose [44]. Even though drugs such as voglibose, acarbose, and miglitol are commercially available as those enzymes’ inhibitors and are also used in practice, they produce undesirous effects such as abdominal discomfort, bloating, and diarrhoea.

In addition, many chronic diseases such as diabetes are associated with oxidative stress, during which reactive oxygen species (O_2_^−^, H_2_O_2_ and OH^−^) are generated. The role of free radicals in the onset and development of diabetes has also been established. Therefore, compounds that possess both antidiabetic and antioxidant properties without causing serious side effects would be of great value [45].

In the present investigation, all extracts were found to inhibit both carbohydrate-hydrolysing enzymes (Amylase: 0.35–2.54 mmol ACAE/g; glucosidase: 0.93–2.20 mmol ACAE/g). Remarkably, the NADES extracts were found to be better inhibitors of amylase and glucosidase compared the other extracts (Figure 3). This could be due to the higher TPC in the NADES extracts, and this fact was also confirmed by Pearson’s correlation analysis (Figure 3). In fact, it has been previously suggested that phenolics are involved in the modulation of the activity of starch digestive enzymes [46].

### 2.5. Data Mining

**Figure 4:**
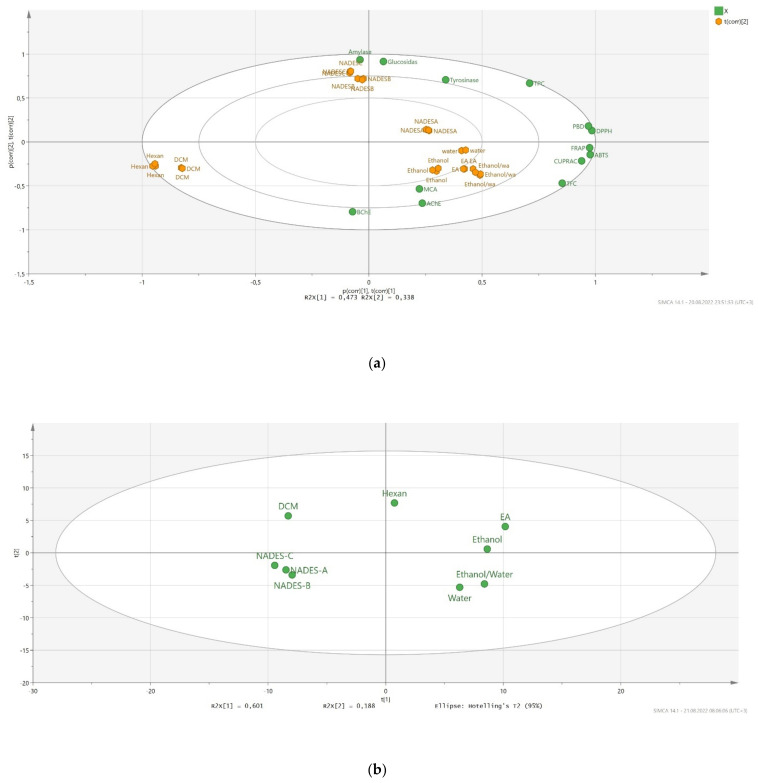
Principal component analysis between tested extracts and biological activities (**a**). Distribution of the tested extracts in principal component analysis by using chemical compound peak areas (**b**).

To gain more insight into the tested extracts and biological activity assays, we performed PCA analysis. The results are given in Figure 4. Firstly, we examined the relationship between the tested extracts based on the biological activity results. We obtained a good distribution, and the tested extracts were very well-separated based on the biological activity results. Two components (PC1: 47.3% and PC2: 33.8) accounted for 811% of the total components. Two extracts (hexane and dichloromethane) exhibited the lowest biological abilities and were distributed in the same axis. In addition, polar extracts (ethanol, ethanol/water, water) and NADES-A had similar biological abilities and were placed in the same group. The PCA plot also confirmed a strong correlation between total flavonoid and antioxidant properties, which were very close to each other on the PCA plot. In addition to biological activity results, we investigated the similarities/differences of the tested extracts based on their chemical profiles. Two components were used in the analysis to determine the distribution of the tested solvents (PC1: 60.1% and PC2: 18.8%). In Figure 4b, the used nonpolar and polar solvents and NADESs were clearly separated, and these results were very similar to the distribution from the biological activity results. Taken together, we concluded that there is a good connection between chemical compounds and biological activities of *Cytinus* extracts.

## 3. Materials and Methods

### 3.1. Materials

The investigated DESs were prepared from reagents of >99% purity (Sigma Aldrich, Burlington, VT, USA), while hydrochloric and sulphuric acid for DES protonation were of analytical reagent grade (POCH, Gliwice, Poland).

### 3.2. Apparatus

DES: chemicals were precisely prepared on a weight basis using a AS.310.R2 analytical balance (Radwag, Radom, Poland). A 06-MSH-PRO-T magnetic stirrer (Chemland, Stargard, Poland) was used to prepare DESs.

### 3.3. Preparation of NADESs

Three NADESs having different properties were used. NADES-A—choline chloride—urea 1:2; NADES-B protonated by HCl L-proline-xylitol 5:1; NADES-C, protonated by H_2_SO_4_ L-proline-xylitol 5:1.

Two synthesis routes were used.

NADES-A was prepared by simple mixing of compounds at 60 °C. The synthesis of the deep eutectic solvents based on protonated L-proline (NADES-A and -B) involved dissolving L-proline in an acid solution (the amount of acid with respect to L-proline was equimolar). Next, a predetermined amount of xylitol was added to the solution. Thus, prepared solution was placed in a rotary evaporator model Rotavapor R-300 (Buchi, Flawil, Switzerland) and water was distilled off under reduced pressure. Studies on the synthesis of this type of DESs and their physicochemical characteristics were the subject of a separate paper [47].

### 3.4. Plant Material and Preparation of Extracts

*Cytinus hypocistis* samples were collected at the flowering season in June 2020 (Anamur, Mersin, Turkey). The plants were identified by one of the authors (Dr. Evren Yildiztugay) and voucher specimens were deposited at the herbarium of Selcuk University, Konya, Turkey. The samples’ aerial parts were dried in the shade at room temperature for about 7 days, and then ground into a powder using a mill. All of the samples were kept in a dark place.

In this study, the extracts were prepared using traditional solvents (n-hexane, ethyl acetate, dichloromethane, ethanol, ethanol/water (70%), and water) and NADEs. Maceration was employed as the extraction method to obtain n-hexane, ethyl acetate, dichloromethane, EtOH, and EtOH/water extracts. The plant materials (10 g) were macerated overnight at room temperature with 200 mL of these solvents. Finally, the solvents were evaporated from the mixtures. To obtain water extracts, the plant materials (10 g) were kept with 200 mL of boiled water, and then the extracts were filtered and lyophilised. In the preparation of NADE extracts, the plant materials (10 g) were mixed with the NADESs for 20 min at 25 °C in an ultrasonic bath. The extracts were filtered and all extracts were stored at 4 °C until further analysis was required.

### 3.5. Chemical Reagents

All chemicals were of HPLC-MS grade and used as received. Acetic acid and acetonitrile for UPLC were purchased from Fluka (Sigma-Aldrich, Steinheim, Germany) and Lab-Scan (Gliwice, Sowinskiego, Poland), respectively. For solutions, ultrapure water was obtained with a Milli-Q system Millipore (Bedford, MA, USA), and absolute ethanol was purchased from VWR chemicals (Radnor, PA, USA).

### 3.6. UPLC-ESI-QTOF-MS Conditions

*Cytinus* extracts were redissolved at 5 mg/mL in the same extraction solvent and filtered by 0.22 µm. The compounds were separated using an ACQUITY UPLC H-Class System (Waters Corp., Milford, MA, USA) with a reversed-phase column (ACQUITY UPLC BEH Shield RP18, 130Å, 1.7 µm, 2.1 mm × 150 mm) at a flow rate of 0.7 mL/min and using a injection volume of 10 μL. The mobile phases were acidified water (0.5% acetic acid, *v*/*v*) and acetonitrile as solvents A and B, respectively. The following multi-step linear gradient was used in order to achieve an efficient separation: 0.00 min [A:B 99/1], 2.33 min [A:B 99/1], 4.37 min [A:B 93/7], 8.11 min [A:B 86/14], 12.19 min [A:B 76/24], 15.99 min [A:B 60/40], 18.31 min [A:B 2/98], 21.03 min [A:B 2/98], 22.39 min [A:B 99/1] and 25.00 [A:B 99/1].

The UPLC was coupled to an electrospray-quadrupole-time of flight mass spectrometer (ESI-QTOF-MS) Synapt G2 (Waters Corp., Milford, MA, USA) working in negative-ionisation mode in a *m*/*z* range from 50 to 1200 *m*/*z*. The MS acquisition was based on two parallel scan functions switching between them continuously. The first function was operated at low collision energy in the gas cell (4 eV) and the other at an elevated collision energy (MS^E^ energy linear ramp: from 20 to 60 eV). Leu-enkephalin was injected for mass calibration continuously. Other MS parameters were, as follows, source temperature 100 °C; scan duration 0.1 s; resolution 20,000 FWHM; desolvation temperature 500 °C; desolvation gas flow 700 L/h; capillary voltage 2.2 kV; cone voltage 30 V; cone gas flow 50 L/h. Finally, the acquired data were processed using MZmine 2.53 open-source software and Sirius 4.4.29.

### 3.7. UPLC-ESI-QTOF-MS Data Processing

Firstly, the raw data files were transformed to. mzML format using MSConvert software. The converted data were processed using the open-source software MZmine 2.53 (Pluskal et al., 2020). A noise level of 1.0 × 10^3^ was selected. ADAP chromatogram builder method was used under the following parameters: MS level: 1; min number of scans: 9; group intensity threshold: 1.0 × 10^3^; min highest intensity: 1.0 × 10^4^; *m*/*z* tolerance: 10 ppm. After that, the chromatogram was deconvoluted using the Wavelets (ADAP) algorithm and the following parameters: S/N threshold: 50; min feature height: 5E4; coefficient/area threshold: 110; peak duration range: 0.05–0.3 min; RT wavelet range: 0–0.30. An isotopic peak grouper algorithm was also applied (*m*/*z* tolerance: 10 ppm, RT tolerance: 0.02 min, maximum charge: 2). The obtained features were aligned between samples using the “Join Aligner” algorithm using a *m*/*z* tolerance of 10 ppm and a RT tolerance of 0.1 min. The molecular features, which were also detected in blank samples, were filtered from the final dataset. Finally, the molecular formulas of the final features were predicted using Sirius 4.4.29 (Dührkop et al., 2019) and the biological identities were annotated by comparing the MS/MS spectra of different databases (e.g., MoNA, Massbank, HMDB, FoodDB, etc.), with the fragments detected in the MSE scans.

### 3.8. Determination of Total Polyphenol and Flavonoids Contents

Total phenolic and flavonoid contents were calculated with the Folin-Ciocalteu and AlCl_3_ assays, respectively [48]. Gallic acid equivalents (mg GAEs/g dry extract) and rutin equivalents (mg REs/g dry extract) were used to describe the outcomes of the two tests.

### 3.9. Antioxidant and Enzyme Inhibitory Assays

The antioxidant and enzyme inhibitory activity of comfrey root extracts was assessed according to methods presented previously [49,50]. Data were expressed as: mg Trolox equivalents (TE)/g extract in ferric ion reducing antioxidant power (FRAP), cupric ion reducing antioxidant capacity (CUPRAC), ABTS and DPPH radical scavenging activity; mg EDTA equivalents (EDTAE)/g extract in the metal chelating ability (MCA), mmol TE/g extract in the phosphomolybdenum assay (PBD); mg galanthamine equivalents (GALAE)/g extract in AChE and BChE assays; mg kojic acid equivalents (KAE)/g extract in tyrosinase inhibitory assay; and mmol acarbose equivalents (ACAE)/g extract in amylase and glucosidase assays.

### 3.10. Data Analysis

All analyses were performed in triplicate and results were reported as means ± SD. Pearson’s correlation coefficients were calculated between total bioactive components and biological activity parameters. Pearson’s correlation was performed by GraphPad version 9.0. The relationship between species, chemical compounds and bioactivities was also assessed using principal component analysis (PCA). PCA analysis was performed by SIMCA version 14.0.

## 4. Conclusions

In the present study, nine extracts of *C. hypocistis* obtained using traditional solvents and NADESs were investigated for their total polyphenolic contents, antioxidant and enzyme inhibitory properties. The extracts were found to be richer in TPC than TFC. In particular, the NADES extracts were found to yield higher TPC compared to the other extracts. On the other hand, generally, ethanol/water, ethanol, and water showed very potent and better antioxidant potential compared to the other extracts, whereas hexane and dichloromethane exhibited weaker antioxidant potential in almost all antioxidant assays. While NADES-A extract displayed the highest anti-AChE activity, none of the NADES extracts displayed inhibition against the butylcholinesterase. With the exception of the water extract, all the other traditional solvent extracts showed dual cholinesterase inhibitory properties. Remarkably, the NADES extracts were found to have an enhanced antityrosinase effect compared to the traditional solvent extracts. Similarly, the NADES extracts were found to be better glucosidase inhibitors, and the NADES-B and -C extracts showed higher antiamylase activity in comparison with the other studied extracts. It is worth highlighting that this study demonstrated NADESs to have brought some improved abilities in bioactive content yields and bioactivity compared to the traditional extracts, especially in terms of TPC and some of the enzyme inhibitory activities. However, the traditional solvents were much better in extracting the antioxidant compounds. Hence, the current investigation enabled a comparison between traditional solvents and NADESs and suggested the potential of NADESs as an alternative to traditional organic solvents for higher extraction of phytonutrients and some better biological performance, although the traditional solvents were found to be more effective in yielding higher antioxidant activity. The paper proved the importance of natural medicine where sources of pro-health components are taken from plants. NADESs as mixtures obtained from compounds of natural origin, in respect to the studied examples, are nontoxic; thus, their extracts could be used as components of functional foods as well as food additives without the need of NADES removal.

## Figures and Tables

**Table 1:** Chemical characterisation of the tested extracts.

Peak	Compound	Hexane	Ethyl Acetate	Dichloromethane	Ethanol	Ethanol/Water	Water	NADES-A	NADES-B	NADES-C
1	Hydroxy-pseudouric acid	+	+	+	ND	+	+	ND	+	ND
2	Galloyl-galactarolactone	+	+	ND	ND	+	+	ND	ND	ND
3	Unknown 1	+	+	+	+	+	+	+	+	+
4	Unknown 2	ND	ND	ND	ND	ND	ND	ND	+	ND
5	Proline	ND	ND	ND	ND	ND	ND	+	ND	+
6	Disaccharide	+	+	ND	+	+	+	ND	ND	ND
7	Unknown 3	ND	ND	ND	ND	ND	ND	ND	ND	+
8	Glucose	ND	ND	ND	+	+	ND	ND	ND	ND
9	Unknown 4	ND	ND	ND	ND	ND	ND	ND	ND	+
10	Quinic acid	ND	+	ND	+	+	+	ND	ND	ND
11	Galloyl-diglucose	ND	ND	ND	+	+	ND	ND	ND	ND
12	Galloylglucose isomer 1	+	+	ND	+	+	+	ND	+	ND
13	Galloylglucose isomer 2	+	+	ND	+	+	+	ND	+	ND
14	Galloylglucose isomer 3	+	+	ND	+	+	+	ND	+	ND
15	Pyrogallol	ND	+	ND	+	+	+	+	+	+
16	Gallic acid	ND	+	ND	+	+	+	+	+	+
17	Galloylglucose isomer 4	ND	+	ND	+	+	+	ND	+	ND
18	Fukiic acid	ND	+	ND	ND	ND	+	ND	+	ND
19	Digalloylglucose isomer 1	ND	+	ND	+	+	+	ND	+	ND
20	Digalloylglucose isomer 2	ND	ND	ND	+	+	+	ND	+	ND
21	Digalloylglucose isomer 3	ND	+	ND	+	+	+	ND	+	ND
22	Digalloylglucose isomer 4	ND	+	ND	+	+	+	ND	+	ND
23	Brevifolin carboxylic acid	ND	ND	ND	ND	+	+	ND	ND	ND
24	Digallate isomer 1	ND	ND	ND	+	+	+	ND	ND	ND
25	Unknown 5	+	+	ND	ND	ND	ND	ND	ND	ND
26	Trigalloyl-glucoside isomer 1	ND	+	ND	+	+	+	+	ND	ND
27	HHDP-galloylglucose isomer 1	ND	+	ND	+	+	+	ND	ND	ND
28	Galloylnorbergenin isomer 1	+	+	ND	+	+	+	+	ND	ND
29	Brevifolin	ND	ND	ND	+	+	+	+	ND	ND
30	Galloylnorbergenin isomer 2	ND	+	ND	+	+	+	ND	ND	ND
31	Digalloyl-HHDP-glucose isomer 1	ND	ND	ND	+	+	+	ND	ND	ND
32	Galloylnorbergenin isomer 3	+	+	ND	+	+	+	ND	ND	ND
33	Trigalloyl-glucoside isomer 2	ND	+	ND	+	+	+	ND	+	ND
34	HHDP-galloylglucose isomer 2	ND	+	ND	+	+	+	ND	ND	ND
35	Trigalloyl-glucoside isomer 3	ND	+	ND	+	+	+	ND	+	ND
36	Digalloyl-HHDP-glucose isomer 2	ND	+	ND	+	+	+	ND	ND	ND
37	HHDP-galloylglucose isomer 3	ND	+	ND	+	+	+	ND	ND	ND
38	Trigalloyl-glucoside isomer 4	ND	+	ND	+	+	+	ND	ND	ND
39	Terflavin B isomer 1	ND	+	ND	+	ND	ND	ND	ND	ND
40	Galloyl-HHDP-glucose isomer 1	ND	+	ND	+	+	+	ND	ND	ND
41	Tetragalloyl-glucoside isomer 1	ND	+	ND	+	+	+	ND	ND	ND
42	Balanophotannin E isomer 1	ND	+	ND	+	+	+	ND	ND	ND
43	Trigalloyl-HHDP-glucose isomer 1	ND	ND	ND	ND	+	ND	ND	ND	ND
44	Terflavin B isomer 2	ND	+	ND	+	ND	ND	ND	ND	ND
45	Galloflavin	+	+	ND	ND	ND	ND	ND	ND	ND
46	Ellagic acid	+	+	ND	+	+	+	+	+	ND
47	Terflavin B isomer 3	+	+	ND	+	ND	ND	ND	ND	ND
48	Trigalloyl-HHDP-glucose isomer 2	+	+	ND	+	+	+	ND	ND	ND
49	Digalloyl-lactonised valoneoyl-d-glucose isomer 1	+	+	ND	+	ND	ND	ND	ND	ND
50	Trigalloyl-brevifolincarboxyl-glucose isomer 1	ND	+	ND	+	+	+	ND	ND	ND
51	Tetragalloyl-glucoside isomer 2	+	+	ND	+	+	+	+	ND	+
52	Catechin	ND	+	ND	+	+	+	ND	ND	ND
53	Tetragalloyl-glucoside isomer 3	+	+	ND	+	+	+	+	ND	+
54	Digallate isomer 2	ND	+	ND	+	+	+	ND	ND	ND
55	Tetragalloyl-glucoside isomer 4	+	+	ND	+	+	+	+	ND	ND
56	Digallate isomer 3	ND	+	ND	+	+	+	ND	ND	ND
57	Terflavin B isomer 4	+	+	ND	+	+	ND	ND	ND	ND
58	Galloyl-HHDP-glucose isomer 2	+	+	ND	+	+	+	+	ND	ND
59	Tetragalloyl-glucoside isomer 5	+	+	ND	+	+	+	+	ND	ND
60	Neochebulagic acid isomer 1	ND	+	ND	+	+	+	ND	ND	ND
61	Neochebulagic acid isomer 2	ND	+	ND	+	+	+	ND	ND	ND
62	Isorhamnetin glucoside isomer 1	+	+	ND	+	+	+	ND	ND	ND
63	Epicatechin	ND	+	ND	+	+	+	ND	ND	ND
64	Quercetin	ND	+	ND	ND	+	ND	ND	ND	ND
65	Balanophotannin E isomer 2	ND	+	ND	+	+	+	ND	ND	ND
66	Unknown 6	+	+	ND	+	+	+	ND	ND	ND
67	Digalloyl-lactonised valoneoyl-d-glucose isomer 2	ND	+	ND	+	ND	ND	ND	ND	ND
68	Trigalloyl-brevifolincarboxyl-glucose isomer 2	+	+	ND	+	+	+	ND	ND	ND
69	Pentagalloyl-glucose isomer 1	+	+	ND	+	+	+	ND	ND	ND
70	Neochebulagic acid isomer 3	ND	ND	ND	ND	+	ND	ND	ND	ND
71	Trigalloyl-lactonised valoneoyl glucose isomer 1	ND	+	ND	+	+	ND	ND	ND	ND
72	(Galloyl)galloyl-tetragalloylglucose isomer 1	ND	+	ND	+	+	+	ND	ND	ND
73	Trigalloyl-brevifolincarboxyl-glucose isomer 3	+	+	ND	+	+	+	ND	ND	ND
74	Trigalloyl-DHHDP-glucose isomer 1	+	+	ND	ND	ND	ND	ND	ND	ND
75	Digalloyl-lactonised valoneoyl-d-glucose isomer 3	+	+	ND	+	+	+	+	ND	ND
76	Castalagin	+	+	ND	ND	ND	ND	ND	ND	ND
77	Trigalloyl-HHDP-glucose isomer 3	+	+	ND	+	+	+	+	ND	ND
78	Galloyl-penta-hydroxy-benzoic-brevifolincarboxyl-glucose isomer 1	+	+	ND	+	+	+	+	ND	ND
79	Pentagalloyl-glucose isomer 2	+	+	ND	+	+	+	+	ND	ND
80	Trisgalloyl HHDP glucose isome isomer 1	+	+	ND	ND	ND	ND	ND	ND	ND
81	Amurensisin	+	+	ND	+	+	+	ND	ND	ND
82	Trigalloyl-DHHDP-glucose isomer 2	ND	+	ND	+	ND	+	ND	ND	ND
83	Digalloyl-lactonised valoneoyl-d-glucose isomer 4	+	+	ND	+	ND	ND	ND	ND	ND
84	Pentagalloyl-glucose isomer 3	+	+	ND	+	+	+	+	ND	ND
85	Galloyl-penta-hydroxy-benzoic-brevifolincarboxyl-glucose isomer 2	+	+	ND	+	+	+	ND	ND	ND
86	Trigalloyl-brevifolincarboxyl-glucose isomer 4	+	+	ND	+	+	ND	ND	ND	ND
87	Phyllanthusiin C isomer 1	+	+	ND	+	+	+	ND	ND	ND
88	Trigalloyl-DHHDP-glucose isomer 3	+	+	ND	ND	+	+	ND	ND	ND
89	Ethyl gallate	ND	ND	ND	+	+	+	ND	ND	ND
90	Trigalloyl-brevifolincarboxyl-glucose isomer 5	+	+	ND	+	+	+	ND	ND	ND
91	Trisgalloyl HHDP glucose isome isomer 2	ND	+	ND	ND	ND	+	ND	ND	ND
92	Trigalloyl-lactonised valoneoyl glucose isomer 2	+	+	ND	+	ND	ND	ND	ND	ND
93	(Galloyl)galloyl-tetragalloylglucose isomer 2	+	+	ND	+	+	+	+	ND	ND
94	Digalloyl-HHDP-iso DHDG-glucose isomer 1	+	+	ND	+	ND	ND	ND	ND	ND
95	Balanophotannin E isomer 3	ND	+	ND	+	+	ND	ND	ND	ND
96	Isorhamnetin glucoside isomer 2	ND	+	ND	+	+	ND	ND	ND	ND
97	Hexagalloyl-glucose isomer 1	+	+	ND	+	+	+	+	ND	ND
98	Trigalloyl-lactonised valoneoyl glucose isomer 3	+	+	ND	+	+	ND	ND	ND	ND
99	Ellagic acid derivative	+	+	ND	ND	ND	+	ND	ND	ND
100	(Galloyl)galloyl-tetragalloylglucose isomer 3	+	+	ND	+	+	+	ND	ND	ND
101	(Galloyl)galloyl-tetragalloylglucose isomer 4	+	+	ND	+	+	+	ND	ND	ND
102	Galloyl-penta-hydroxy-benzoic-brevifolincarboxyl-glucose isomer 3	ND	+	ND	+	+	+	ND	ND	ND
103	Hexagalloyl-glucose isomer 2	+	+	ND	+	+	+	+	ND	ND
104	Galloyl-HHDP-glucose isomer 3	+	+	ND	+	+	+	+	ND	ND
105	Hexagalloyl-glucose isomer 3	+	+	ND	+	+	+	+	ND	ND
106	Digalloyl-HHDP-iso DHDG-glucose isomer 2	ND	+	ND	ND	+	+	ND	ND	ND
107	Tetragalloyl-hydroxybenzoyl-glucopyranoside isomer 1	ND	+	ND	+	+	ND	ND	ND	ND
108	Heptagalloyl hexose isomer 1	+	+	ND	+	+	+	ND	ND	ND
109	Galloylmyricetin	+	+	ND	+	+	+	+	ND	ND
110	Heptagalloyl hexose isomer 2	+	+	ND	+	+	+	+	ND	ND
111	Phyllanthusiin C isomer 2	ND	+	ND	ND	+	+	ND	ND	ND
112	Digalloyl-HHDP-iso DHDG-glucose isomer 3	ND	+	ND	ND	+	+	ND	ND	ND
113	Trigalloyl-brevifolincarboxyl-glucose isomer 6	ND	+	ND	+	+	ND	ND	ND	ND
114	Heptagalloyl hexose isomer 3	+	+	ND	+	+	+	ND	ND	ND
115	Tetragalloyl-hydroxybenzoyl-glucopyranoside isomer 2	+	+	ND	+	+	+	ND	ND	ND
116	Tetragalloyl-hydroxybenzoyl-glucopyranoside isomer 3	+	+	ND	+	ND	+	ND	ND	ND
117	Unknown 7	ND	ND	ND	+	ND	ND	ND	ND	ND
118	Trihydroxy-octadecenoic acid	ND	+	+	ND	ND	ND	ND	ND	ND
119	Hydroxyretinoic acid	+	ND	+	ND	ND	ND	ND	ND	ND
120	Unknown 8	+	+	+	ND	ND	ND	ND	ND	ND
121	Hexadecanedioic acid	+	+	+	ND	ND	ND	ND	ND	ND
122	Hydroxyeicosatrienoic acid	+	+	+	+	ND	ND	ND	ND	ND
123	Valerenic acid	+	+	+	+	ND	ND	ND	ND	ND
124	Hydroxylinoleic acid	+	+	+	ND	ND	ND	ND	ND	ND
125	Linoleic acid	ND	+	+	+	ND	ND	ND	ND	ND
126	Hydroxylinolenic acid	+	+	+	+	+	ND	ND	ND	ND
127	Dodecenyl-succinic anhydride	+	+	+	+	ND	ND	ND	ND	ND
128	Retinoic acid	+	+	+	ND	ND	ND	ND	ND	ND
129	Oleic acid	+	+	+	+	+	ND	ND	+	ND
130	Pentadecenoic acid	+	+	+	+	+	ND	ND	ND	ND
131	Oleiyl glucoside	ND	+	+	ND	ND	ND	ND	ND	ND
132	Linolenic acid	+	+	+	+	+	+	ND	ND	ND
133	Stearic acid	+	+	ND	+	ND	ND	ND	ND	ND
134	Unknown 9	+	+	+	+	+	ND	ND	ND	ND
135	Unknown 10	+	+	+	+	ND	ND	ND	ND	ND
136	Eicosapentaenoic acid	+	+	+	+	ND	+	ND	ND	ND
137	Palmitoleic acid	+	+	+	ND	+	+	+	ND	+
138	Linoleic acid	+	+	+	+	+	+	ND	ND	+
139	Oxodecanedioic acid	+	ND	ND	ND	ND	ND	ND	ND	ND
140	Methyl arachidonate	+	+	+	+	ND	ND	ND	ND	ND
141	Arjungenin	ND	ND	+	ND	ND	ND	ND	ND	ND
142	Heptadecenoic acid	+	ND	ND	ND	+	+	ND	ND	ND
143	Unknown 11	ND	+	+	+	ND	ND	ND	ND	ND
144	Unknown 12	+	+	+	+	+	+	ND	ND	ND
145	Glycerylmonooleate	+	ND	ND	ND	ND	ND	ND	ND	ND
146	Palmitic acid	+	ND	ND	+	+	+	+	+	+
147	Hydroxydocosanoic acid	+	+	+	+	+	ND	ND	ND	ND
148	Dodecenylsuccinic acid	ND	+	ND	ND	+	ND	ND	ND	ND

+: present; ND: nondetected.

## Data Availability

Not applicable.

## Supplementary Materials

The following supporting information can be downloaded at: https://www.mdpi.com/article/10.3390/molecules27185788/s1, Table S1: Total phenolic (TPC) and flavonoid content (TFC) of the tested extracts; Table S2: Proposed annotated compounds by UPLC-ESI-QTOF-MS in all Cytinus extracts; Table S3: Compound areas extracted for each Cytinus extract; Table S4: Antioxidant properties of the tested extracts; Table S5: Enzyme inhibitory of the tested extracts.
