# Supplementary material for: Selectivity Tuning by Natural Deep Eutectic Solvents (NADESs) for Extraction of Bioactive Compounds from Cytinus hypocistis—Studies of Antioxidative, Enzyme-Inhibitive Properties and LC-MS Profiles
Source: Molecules. 2022 Sep 7;27(18):5788. doi: 10.3390/molecules27185788 (PMC9502194; doi:10.3390/molecules27185788)
Supplement: Supplementary file 1 [file molecules-27-05788-s001.zip › molecules-1904677-supplementary.pdf]

Article

Selectivity tuning by natural deep eutectic solvents (NADES) for extraction of bioactive compounds from *Cytinus hypocistis* – studies of antioxidative and enzyme inhibitive properties and LC-MS profiles

Gokhan Zengin[1], María de la Luz Cádiz-Gurrea[2*], Álvaro Fernández-Ochoa[2*], Francisco Javier Leyva-Jiménez[3,4] Malwina Momotko[5], Evren Yildiztugay[6], Refik Karatas[1], Sharmeen Jugreet[7], Mohamad Fawzi Mahoomodally[7,8,9], Grzegorz Boczkaj[10,11]

[1]Department of Biology, Science Faculty, Selcuk University, Konya, Turkey
[2]Department of Analytical Chemistry, Faculty of Sciences, University of Granada, Fuentenueva s/n, E- 18071 Granada, Spain
[3]Department of Analytical Chemistry and Food Science and Technology, University of Castilla-La Mancha, Ronda de Calatrava 7, 13071, Ciudad Real, Spain
[4]Regional Institute for Applied Scientific Research (IRICA), Area of Food Science, University of Castilla-La Mancha, Avenida Camilo Jose Cela, 10, 13071, Ciudad Real, Spain
[5]Gdansk University of Technology, Faculty of Chemistry, Department of Process Engineering and Chemical Technology, 80 – 233 Gdansk, G. Narutowicza St. 11/12, Poland
[6]Department of Biotechnology, Science Faculty, Selcuk University, Konya, Turkey
[7] Department of Health Sciences, Faculty of Medicine and Health Sciences, University of Mauritius, Réduit 80837, Mauritius
[8] Center for Transdisciplinary Research ,Department of Pharmacology ,Saveetha Dental College ,Saveetha Institute of Medical and Technical Science ,Chennai ,India
[9]Centre of Excellence for Pharmaceutical Sciences (Pharmacen), North West University, South Africa
[10]Gdansk University of Technology, Faculty of Civil and Environmental Engineering, Department of Sanitary Engineering, 80 – 233 Gdansk, G. Narutowicza St. 11/12, Poland
[11]Advanced Materials Center, Gdansk University of Technology, 80 – 233 Gdansk, G. Narutowicza St. 11/12, Poland
* Correspondence: mluzcadiz@ugr.es; alvaroferochoa@ugr.es

Table S1. Total phenolic (TPC) and flavonoid content (TFC) of the tested extracts.

**Solvents**	**TPC (mg GAE/g)**	**TFC (mg RE/g)**
Hexane	26.47±0.96^{g}	0.68±0.02^{g}
Ethyl acetate	127.83±1.93^{d}	12.55±0.35^{a}
Dichloromethane	43.28±0.06^{f}	1.85±0.01^{f}
Ethanol	123.68±0.59^{e}	8.82±0.44^{c}
Ethanol/water	123.51±0.86^{e}	11.44±0.14^{b}
Water	126.56±1.47^{de}	9.35±0.18^{c}
NADES-A	174.67±0.62^{b}	5.89±0.15^{d}
NADES-B	186.13±1.84^{a}	3.82±0.06^{e}
NADES-C	167.57±1.45^{c}	1.51±0.08^{f}

Values are reported as mean ±SD of three parallel experiments. GAE: Gallic acid equivalent; RE: Rutin equivalent. Different letters in same column indicate significant differences in the tested extracts ($p<0.05$)

Table S2. Proposed annotated compounds by UPLC-ESI-QTOF-MS in all Cytinus extracts.

Peak	RT (min)	m/z	m/z theorical	Error (ppm)	Formula	Proposed identification of the compounds	MS Fragments
1	0.46	201.0248	201.0265	-8.86	C5H6N4O5	Hydroxy-pseudouric acid	201.0248
2	0.47	343.0364	343.0307	16.54	C13H12O11	Galloyl-galactarolactone	343.0364
3	0.47	217.0023	217.0006	7.71	C4H5N5O4P	Unknown 1	---
4	0.61	222.1344	222.1347	-1.15	C9H21NO5	Unknown 2	---
5	0.64	114.0555	114.0561	-4.90	C5H9NO2	Proline	114.0555
6	0.66	341.1083	341.1089	-1.79	C12H22O11	Disaccharide	101.0265/179.0698
7	0.67	328.0698	328.0674	7.43	C13H15NO9	Unknown 3	---
8	0.67	179.0557	179.0561	-2.18	C6H12O6	Glucose	179.0557
9	0.71	231.0172	231.0180	-3.72	C5H12O8S	Unknown 4	---
10	0.71	191.0552	191.0561	-4.95	C7H12O6	Quinic acid	85.0231/93.0368
11	0.76	493.1202	493.1199	0.70	C19H26O15	Galloyl-diglucose	125.0312/169.0213/493.1202
12	0.79	331.0666	331.0671	-1.50	C13H16O10	Galloylglucose isomer 1	125.0312/169.0213
13	1.09	331.0661	331.0671	-2.85	C13H16O10	Galloylglucose isomer 2	125.0247/169.0160
14	1.32	331.0660	331.0671	-3.17	C13H16O10	Galloylglucose isomer 3	125.0244/169.0147
15	1.64	125.0235	125.0244	-6.98	C6H6O3	Pyrogallol	125.0235
16	1.65	169.0137	169.0142	-3.17	C7H6O5	Gallic acid	125.0231/169.0137
17	1.78	331.0662	331.0671	-2.64	C13H16O10	Galloylglucose isomer 4	169.0128/331.0662
18	1.89	271.0458	271.0459	-0.44	C11H12O8	Fukiic acid	271.0458
19	4.35	483.0775	483.0780	-1.18	C20H20O14	Digalloylglucose isomer 1	125.0240/169.0130/331.0633
20	4.70	483.0769	483.0780	-2.28	C20H20O14	Digalloylglucose isomer 2	125.0228/169.0113/313.0548/331.0644
21	5.11	483.0775	483.0780	-1.18	C20H20O14	Digalloylglucose isomer 3	125.0266/169.0122/331.0709
22	5.96	483.0774	483.0780	-1.28	C20H20O14	Digalloylglucose isomer 4	125.0236/169.0122/313.0616/331.0616
23	6.17	291.0140	291.0146	-2.32	C13H8O8	Brevifolin carboxylic acid	191.0273/247.0242
24	6.39	321.0248	321.0252	-1.16	C14H10O9	Digallate isomer 1	125.0238/169.0127
25	6.82	601.0463	601.0530	-11.08	C19H22O22	Unknown 5	---

26	6.83	635.0876	635.0890	-2.20	C27H24O18	Trigalloyl-glucoside isomer 1	107.0138/125.0246/169.0147/465.0677
27	6.84	633.0715	633.0733	-2.97	C27H22O18	HHDP-galloylglucose isomer 1	125.0251/169.0129/300.9979/463.0687
28	6.84	465.0660	465.0675	-3.11	C20H18O13	Galloylnorbergenin isomer 1	125.0229/169.0125
29	7.39	247.0239	247.0248	-3.59	C12H8O6	Brevifolin	191.0313/219.0316
30	7.49	465.0669	465.0675	-1.11	C20H18O13	Galloylnorbergenin isomer 2	125.0228/169.0122
31	7.81	785.0836	785.0843	-0.82	C34H26O22	Digalloyl-HHDP-glucose isomer 1	169.0134/301.0093/483.0655
32	7.91	465.0671	465.0675	-0.88	C20H18O13	Galloylnorbergenin isomer 3	125.0230/169.0127
33	7.91	635.0890	635.0890	-0.04	C27H24O19	Trigalloyl-glucoside isomer 2	125.0235/169.0149/211.0183/313.0580/ 465.0672
34	7.93	633.0734	633.0733	0.15	C27H22O18	HHDP-galloylglucose isomer 2	125.0244/169.0147/249.0323/275.0092/ 301.0023/
35	8.00	635.0885	635.0890	-0.76	C27H24O18	Trigalloyl-glucoside isomer 3	125.0245/169.0143/211.0254/313.0564/ 465.0665
36	8.30	785.0828	785.0843	-1.87	C34H26O22	Digalloyl-HHDP-glucose isomer 2	125.0240/169.0123/300.9958/313.0535/ 463.0874
37	8.39	633.0723	633.0733	-1.64	C27H22O18	HHDP-galloylglucose isomer 3	125.0271/169.0146/300.9971/463.0655
38	8.40	635.0880	635.0890	-1.57	C27H24O19	Trigalloyl-glucoside isomer 4	125.0278/169.0143/313.0576/483.0790
39	8.91	783.0687	783.0686	0.10	C34H24O22	Terflavin B isomer 1	450.9983/631.0520
40	8.93	617.0775	617.0784	-1.55	C27H22O17	Galloyl-HHDP-glucose isomer 1	169.0123/300.9988
41	8.94	787.0977	787.0999	-2.86	C34H28O22	Tetragalloyl-glucoside isomer 1	169.0097/313.0591/465.0709/617.0779/ 635.0878
42	9.18	757.0863	757.0894	-4.05	C33H26O21	Balanophotannin E isomer 1	125.0219/169.0131
43	9.27	937.0925	937.0953	-2.93	C41H30O26	Trigalloyl-HHDP-glucose isomer 1	169..0131/295.0461/300.9969
44	9.28	783.0683	783.0686	-0.44	C34H24O22	Terflavin B isomer 2	783.0663
45	9.39	276.9971	276.9990	-6.65	C12H6O8	Galloflavin	169.0141
46	9.47	300.9977	300.9990	-4.43	C14H6O8	Ellagic acid	145.0327/245.0069/300.9953
47	9.57	783.0671	783.0686	-2.04	C34H24O22	Terflavin B isomer 3	450.9964/631.0720
48	9.57	937.0949	937.0953	-0.36	C41H30O26	Trigalloyl-HHDP-glucose isomer 2	169.0134/275.0200/295.0451/300.9987/ 313.0562/465.0669
49	9.58	935.0806	935.0796	1.01	C41H28O26	Digalloyl-lactonised valoneoyl-d-glucose isomer 1	125.0246/169.0140/300.9976/450.9925

50	9.59	909.0993	909.1003	-1.13	C40H30O25	trigalloyl-brevifolincarboxyl-glucose isomer 1	247.0258/435.0508/
51	9.61	787.0993	787.0999	-0.81	C34H28O22	Tetragalloyl-glucoside isomer 2	125.0241/169.0135/313.0585/465.0690/617.0760/635.0874
52	9.63	289.0708	289.0718	-3.43	C15H14O6	Catechin	245.0830
53	9.67	787.0997	787.0999	-0.31	C34H28O22	Tetragalloyl-glucoside isomer 3	125.0232/169.0134/313.0546/617.0777/635.0831
54	9.70	321.0238	321.0252	-4.34	C14H10O9	Digallate isomer 2	125.0229/169.0133
55	9.76	787.0988	787.0999	-1.43	C34H28O22	Tetragalloyl-glucoside isomer 4	125.0232/169.0140/313.0562/465.0684/617.0783/635.0871
56	9.78	321.0242	321.0252	-3.11	C14H10O9	Digallate isomer 3	125.0238/169.0138
57	9.78	783.0677	783.0686	-1.19	C34H24O22	Terflavin B isomer 4	450.0698/631.0460
58	9.89	617.0776	617.0784	-1.30	C27H22O17	Galloyl-HHDP-glucose isomer 2	169.0141/300.9985
59	9.91	787.0987	787.0999	-1.64	C34H28O22	Tetragalloyl-glucoside isomer 5	125.0238/169.0141/313.0553/465.0697/617.0778/635.0883
60	9.95	971.1000	971.1007	-0.76	C41H32O28	Neochebulagic acid isomer 1	300.9972/467.0727/935.0848/971.1000
61	10.04	971.0992	971.1007	-1.61	C41H32O28	Neochebulagic acid isomer 2	300.9980/467.0734/971.0992
62	10.05	477.1027	477.1039	-2.41	C22H22O12	Isorhamnetin glucoside isomer 1	271.0299/285.0424/317.0688
63	10.06	289.0708	289.0718	-3.18	C15H14O6	Epicatechin	109.0343/123.0090/245.0076
64	10.25	301.0334	301.0354	-6.72	C15H10O7	Quercetin	121.0292/151.0386/179.0370
65	10.26	757.0882	757.0894	-1.61	C33H26O21	Balanophotannin E isomer 2	125.0219/169.0131/757.0882
66	10.26	463.0867	463.0823	9.41	C28H16O7	Unknown 6	---
67	10.27	935.0740	935.0796	-5.99	C41H28O26	Digalloyl-lactonised valoneoyl-d-glucose isomer 2	125.0240/169.0144/301.0026//767.0713
68	10.29	909.0990	909.1003	-1.47	C40H30O25	trigalloyl-brevifolincarboxyl-glucose isomer 2	247.0258/435.0553/605.0803
69	10.30	939.1064	939.1109	-4.83	C41H32O26	Pentagalloyl-glucose isomer 1	125.0228/169.0139/313.0529/465.0643/617.0801
70	10.41	971.1002	971.1007	-0.56	C41H32O28	Neochebulagic acid isomer 3	300.9987/465.0658/935.0884/971.1002
71	10.49	1087.0912	1087.0906	0.58	C48H32O30	Trigalloyl-lactonised valoneoyl glucose isomer 1	125.0228/169.0129/300.9972/615.0730

72	10.49	1089.1055	1089.1062	-0.61	C48H34O30	(Galloyl)galloyl-tetragalloylglucose isomer 1	125.0227/169.0134/447.0501/465.0643/617.0733/769.0824/939.1045
73	10.59	909.0995	909.1003	-0.91	C40H30O25	trigalloyl-brevifolincarboxyl-glucose isomer 3	247.0263/435.0539/605.0590
74	10.65	953.0909	953.0902	0.74	C41H30O27	Trigalloyl-DHHDP-glucose isomer 1	125.0250/169.0134/275.0188/300.9972/445.0373
75	10.66	935.0791	935.0796	-0.57	C41H28O26	Digalloyl-lactonised valoneoyl-d-glucose isomer 3	125.0235/169.0149/300.9918/767.0920
76	10.70	933.0624	933.0640	-1.64	C41H26O26	Castalagin	933.0624
77	10.70	937.0936	937.0953	-1.81	C41H30O26	Trigalloyl-HHDP-glucose isomer 3	169.0125/295.0440/300.9983/313.051/599.0624
78	10.71	941.0959	941.0902	6.05	C41H34O26	Galloyl-penta-hydroxy-benzoic-brevifolincarboxyl-glucose isomer 1	247.0227/291.0133/435.0532/757.0858
79	10.72	939.1108	939.1109	-0.10	C41H32O26	Pentagalloyl-glucose isomer 2	125.0245/169.0139/465.0652/617.0781/769.0894
80	10.75	951.0757	951.0745	1.28	C41H28O27	Trisgalloyl HHDP glucose isome isomer 1	275.0240/300.9964/605.0799/783.0612/951.0757
81	10.82	439.0658	439.0671	-2.84	C22H16O10	Amurensisin	439.0658
82	10.92	953.0899	953.0902	-0.31	C41H30O27	Trigalloyl-DHHDP-glucose isomer 2	125.0234/169.0140/301.0017/785.0820
83	10.92	935.0794	935.0796	-0.18	C41H28O26	Digalloyl-lactonised valoneoyl-d-glucose isomer 4	125.0232/169.0142/300.9996/767.0502
84	10.93	939.1104	939.1109	-0.57	C41H32O26	Pentagalloyl-glucose isomer 3	169.0144/313.0536/465.0673/617.0775/
85	10.94	941.0942	941.0902	-4.25	C40H30O27	Galloyl-penta-hydroxy-benzoic-brevifolincarboxyl-glucose isomer 2	247.0238/291.0046/757.0898
86	11.06	909.1003	909.1003	-0.06	C40H30O25	trigalloyl-brevifolincarboxyl-glucose isomer 4	169.0134/247.0242/291.0148/435.0642/
87	11.23	925.0937	925.0953	-1.64	C40H30O26	Phyllanthusiin C isomer 1	169.0133/247.0226/300.9927/435.0593/925.0937
88	11.28	953.0887	953.0902	-1.52	C41H30O27	Trigalloyl-DHHDP-glucose isomer 3	125.0241/169.0128/301.0095
89	11.29	197.0448	197.0455	-3.77	C9H10O5	Ethyl gallate	169.0131/124.0169/197.0448
90	11.30	909.0991	909.1003	-1.33	C40H30O25	trigalloyl-brevifolincarboxyl-glucose isomer 5	247.0229/291.0147/435.0540/605.0792

91	11.31	951.0737	951.0745	-0.85	C41H28O27	Trisgalloyl HHDP glucose isome isomer 2	301.0014/605.0767/783.0887
92	11.39	1087.0901	1087.0906	-0.40	C48H32O30	Trigalloyl-lactonised valoneoyl glucose isomer 2	125.0228/169.0129/300.9972/615.0730
93	11.44	1089.1045	1089.1062	-1.56	C48H34O30	(Galloyl)galloyl-tetragalloylglucose isomer 2	125.0227/169.0130/447.0501/465.0662/617.0750/769.0837/939.1060
94	11.47	1105.1029	1105.1011	1.58	C48H34O31	Digalloyl-HHDP-iso DHDG-glucose isomer 1	169.0140/275.0172/300.9943/767.0491
95	11.47	757.0888	757.0894	-0.76	C33H26O21	Balanophotannin E isomer 3	125.0219/169.0131/757.0882
96	11.47	477.1029	477.1039	-2.04	C22H22O12	Isorhamnetin glucoside isomer 2	271.0297/285.0422/317.0684
97	11.56	1091.1213	1091.1219	-0.47	C48H36O30	Hexagalloyl-glucose isomer 1	169.0143/431.0576/617.0773/769.0881/939.1062
98	11.56	1087.0894	1087.0906	-1.05	C48H32O30	Trigalloyl-lactonised valoneoyl glucose isomer 3	125.0219/169.0133/300.9963
99	11.60	299.9898	299.9912	-4.58	C14H5O8	Ellagic acid derivative	299.9898
100	11.63	1089.1052	1089.1062	-0.96	C48H34O30	(Galloyl)galloyl-tetragalloylglucose isomer 3	125.0227/169.0142/447.0501/465.0662/617.0765/769.0887/939.1076
101	11.68	1089.1068	1089.1062	0.51	C48H34O30	(Galloyl)galloyl-tetragalloylglucose isomer 4	125.0227/169.0149/447.0501/465.0662/617.0771/769.0980/939.1094
102	11.69	941.0932	941.0902	-3.18	C40H30O27	Galloyl-penta-hydroxy-benzoic-brevifolincarboxyl-glucose isomer 3	247.0244/291.0181/435.0531/757.0914
103	11.72	1091.1222	1091.1219	0.35	C48H36O30	Hexagalloyl-glucose isomer 2	169.0133/313.0620/617.0771/769.0848/939.1060
104	11.77	617.0779	617.0784	-0.87	C27H22O17	Galloyl-HHDP-glucose isomer 3	169.0134/300.9969
105	11.86	1091.1223	1091.1219	0.38	C48H36O30	Hexagalloyl-glucose isomer 3	169.0128/313.0544/483.0786/617.0781/787.0980/939.1072
106	12.02	1105.0998	1105.1011	-1.16	C48H34O31	Digalloyl-HHDP-iso DHDG-glucose isomer 2	169.0141/275.0171/300.9980/767.0490
107	12.06	907.1187	907.1211	-2.62	C41H32O24	Tetragalloyl-hydroxybenzoyl-glucopyranoside isomer 1	169.0131/300.9990
108	12.09	1243.1330	1243.1328	0.10	C55H40O34	Heptagalloyl hexose isomer 1	169.0145/617.0778/787.1007/939.1128/1091.1259

109	12.11	469.0511	469.0471	8.55	C15H18O17	Galloylmyricetin	169.0141/469.0511
110	12.18	1243.1329	1243.1328	0.08	C55H40O34	Heptagalloyl hexose isomer 2	169.0141/617.0781/769.0922/939.1141/ 1091.1246
111	12.18	925.0942	925.0953	-1.09	C40H30O26	Phyllanthusiin C isomer 2	169.0141/247.0245/300.9989/755.0680
112	12.25	1105.0996	1105.1011	-1.36	C48H34O31	Digalloyl-HHDP-iso DHDG-glucose isomer 3	169.0140/275.0170/300.9983/767.0493
113	12.27	909.0997	909.1003	-0.71	C40H30O25	trigalloyl-brevifolincarboxyl-glucose isomer 6	247.0229/291.0147/435.0540/605.0792
114	12.30	1243.1334	1243.1328	0.47	C55H40O34	Heptagalloyl hexose isomer 3	169.0147/617.0800/769.0877/939.1129/ 1091.1222
115	12.55	907.1194	907.1211	-1.80	C41H32O24	Tetragalloyl-hydroxybenzoyl-glucopyranoside isomer 2	169.0126/300.9985
116	12.64	907.1194	907.1211	-1.82	C41H32O24	Tetragalloyl-hydroxybenzoyl-glucopyranoside isomer 3	169.0122/300.9987
117	13.97	983.1718	983.1793	-7.62	C37H44O31	Unknown 7	---
118	14.64	329.2325	329.2333	-2.67	C18H34O5	Trihydroxy-octadecenoic acid	329.2325
119	16.82	315.1955	315.1966	-3.54	C20H28O3	Hydroxyretinoic acid	315.1955
120	17.44	277.1798	277.1809	-4.13	C17H26O3	Unknown 8	---
121	17.64	285.2057	285.2071	-4.96	C16H30O4	Hexadecanedioic acid	285.2057
122	17.64	321.2423	321.2435	-3.73	C20H34O3	Hydroxyeicosatrienoic acid	321.2423
123	17.68	233.1537	233.1547	-4.47	C15H22O2	Valerenic acid	233.1537
124	17.77	295.2267	295.2279	-3.80	C18H32O3	Hydroxylinoleic acid	295.2267
125	17.81	279.2320	279.2330	-3.49	C18H32O2	Linoleic acid	279.2320
126	17.87	293.2115	293.2122	-2.43	C18H30O3	Hydroxylinolenic acid	293.2115
127	17.93	265.1795	265.1809	-5.33	C16H26O3	Dodecenyl-succinic anhydride	265.1795
128	18.17	299.2007	299.2017	-3.05	C20H28O2	Retinoic acid	299.2007
129	18.24	281.2477	281.2486	-3.16	C18H34O2	Oleic acid	281.2477
130	18.33	239.2003	239.2017	-5.57	C15H28O2	Pentadecenoic acid	239.2003
131	18.36	443.3010	443.3014	-0.89	C24H44O7	Oleiyl glucoside	443.3010
132	18.36	277.2165	277.2173	-2.93	C18H30O2	Linolenic acid	277.2165
133	18.41	283.2629	283.2643	-4.77	C18H36O2	Stearic acid	283.2629

134	18.41	443.3367	443.3378	-2.58	C25H48O6	Unknown 9	---
135	18.42	447.2741	447.2752	-2.60	C26H40O6	Unknown 10	---
136	18.49	301.2166	301.2173	-2.44	C20H30O2	Eicosapentaenoic acid	301.2166
137	18.50	253.2163	253.2173	-3.89	C16H30O2	Palmitoleic acid	253.2163
138	18.56	279.2320	279.2330	-3.50	C18H32O2	Linoleic acid	279.2320
139	18.58	243.1222	243.1238	-6.41	C12H20O5	Oxodecanedioic acid	243.1222
140	18.62	317.2475	317.2486	-3.46	C21H34O2	Methyl arachidonate	317.2475
141	18.65	503.3379	503.3378	0.24	C30H48O6	Arjungenin	503.3379
142	18.65	267.2321	267.2330	-3.04	C17H32O2	Heptadecenoic acid	267.2321
143	18.66	465.3578	465.3585	-1.51	C28H50O5	Unknown 11	---
144	18.70	471.3681	471.3691	-2.15	C27H52O6	Unknown 12	---
145	18.70	369.2699	369.2646	14.15	C21H38O5	Glycerylmonooleate	369.2699
146	18.76	255.2321	255.2330	-3.27	C16H32O2	Palmitic acid	255.2321
147	18.84	355.3209	355.3218	-2.44	C22H44O3	Hydroxydocosanoic acid	355.3209
148	20.83	283.1909	283.1915	-2.05	C16H28O4	Dodecenylsuccinic acid	283.1909

RT: retention time

Table S3. Compound areas extracted for each *Cytinus* extract.

Peak	HEXANE	ETHYL ACETATE	DICHLOROMETHANE	ETHANOL	ETHANOL/WATER	WATER	NADES A	NADES B	NADES C
1	4338.84	7065.39	1722.18	ND	30270.76	9196.48	ND	4257.48	ND
2	1472.37	2555.67	ND	ND	26658.72	4787.31	ND	ND	ND
3	3701.65	10686.53	3525.34	1193.59	11130.14	9630.11	5258.70	8258.92	6534.77
4	ND	ND	ND	ND	ND	ND	ND	23668.11	ND
5	ND	ND	ND	ND	ND	ND	43410.37	ND	26187.67
6	5529.35	11446.12	ND	5575.87	11922.77	3647.79	ND	ND	ND
7	ND	ND	ND	ND	ND	ND	ND	ND	127404.30
8	ND	ND	ND	3027.69	4149.75	ND	ND	ND	ND
9	ND	ND	ND	ND	ND	ND	ND	ND	79290.61
10	ND	5836.29	ND	2196.18	4929.46	2246.15	ND	ND	ND
11	ND	ND	ND	5409.12	3206.05	ND	ND	ND	ND
12	14467.47	11996.40	ND	188302.61	71834.92	27602.13	ND	15803.16	ND
13	1901.12	61452.56	ND	63652.51	4150ND	31270.43	ND	9826.70	ND
14	18705.91	131115.08	ND	104956.04	105101.51	167287.82	ND	7125.64	ND
15	ND	17787.67	ND	16084.88	25204.94	28606.73	4668.04	7010.09	6979.67
16	ND	7640.96	ND	24611.76	28140.39	35129.83	7661.75	6806.10	4156.76
17	ND	8006.45	ND	6522.83	22798.39	18574.97	ND	2330.38	ND
18	ND	8475.20	ND	ND	ND	7478.05	ND	6556.02	ND
19	ND	4098.23	ND	8596.24	41135.99	36057.78	ND	9140.80	ND
20	ND	ND	ND	4565.72	48566.66	35115.73	ND	2311.39	ND
21	ND	11659.83	ND	21870.97	33172.35	2909.42	ND	4106.42	ND
22	ND	6319.32	ND	18824.92	39406.30	24698.35	ND	5947.75	ND
23	ND	ND	ND	ND	20328.68	6145.20	ND	ND	ND
24	ND	ND	ND	1324.12	14369.48	5911.97	ND	ND	ND
25	1397.47	9276.60	ND	ND	ND	ND	ND	ND	ND
26	ND	58810.84	ND	81787.34	216160.54	142065.89	9038.45	ND	ND

27	ND	18678.25	ND	6843.51	11057.52	14186.62	ND	ND	ND
28	2417.88	33927.38	ND	25050.89	49735.92	46090.20	5170.67	ND	ND
29	ND	ND	ND	3591.30	56844.72	25903.39	2442.57	ND	ND
30	ND	6370.92	ND	11661.74	32055.64	11802.14	ND	ND	ND
31	ND	ND	ND	4987.80	26798.91	1254.77	ND	ND	ND
32	1084.70	33514.03	ND	30934.70	52235.40	30551.04	ND	ND	ND
33	ND	84849.87	ND	125377.26	262852.42	106751.63	ND	1499.45	ND
34	ND	30683.41	ND	11464.54	16052.21	11953.01	ND	ND	ND
35	ND	30780.04	ND	53826.53	102630.93	40428.71	ND	1697.95	ND
36	ND	4528.82	ND	19127.96	35912.21	4328.67	ND	ND	ND
37	ND	7278.28	ND	1689.37	4940.15	3152.29	ND	ND	ND
38	ND	13001.39	ND	32157.16	61366.06	21982.25	ND	ND	ND
39	ND	16659.90	ND	1064.04	ND	ND	ND	ND	ND
40	ND	9175.29	ND	12840.77	13054.64	7980.43	ND	ND	ND
41	ND	53399.17	ND	265818.56	184755.56	77494.68	ND	ND	ND
42	ND	8968.07	ND	31473.02	78504.05	27301.00	ND	ND	ND
43	ND	ND	ND	ND	19202.85	ND	ND	ND	ND
44	ND	12358.48	ND	2328.27	ND	ND	ND	ND	ND
45	16208.14	15082.45	ND	ND	ND	ND	ND	ND	ND
46	22672.55	248841.27	ND	361874.10	115949.04	109713.73	6350.37	2969.21	ND
47	1034.34	13423.75	ND	3265.64	ND	ND	ND	ND	ND
48	5665.86	46970.87	ND	31365.63	44628.25	18129.81	ND	ND	ND
49	1547.67	6704.43	ND	1733.79	ND	ND	ND	ND	ND
50	ND	43400.52	ND	25032.40	5406.55	1051.77	ND	ND	ND
51	30707.11	259404.70	ND	250879.69	1027511.76	342713.83	2612.51	ND	1370.26
52	ND	3846.58	ND	1186.27	4159.85	1630.87	ND	ND	ND
53	80419.98	633350.06	ND	506625.26	707367.44	273110.44	8629.73	ND	1209.28
54	ND	5658.15	ND	1336.08	1957.65	1068.36	ND	ND	ND
55	33815.49	137013.88	ND	354567.80	178583.98	267247.71	15013.78	ND	ND

56	ND	19372.27	ND	14787.16	14535.46	9567.92	ND	ND	ND
57	8336.46	74712.31	ND	36873.82	5089.53	ND	ND	ND	ND
58	13715.91	69829.43	ND	42085.46	66113.87	31639.05	2666.10	ND	ND
59	14794.27	157672.40	ND	122414.62	138787.17	103658.89	2963.01	ND	ND
60	ND	54400.71	ND	8907.23	108740.88	32329.29	ND	ND	ND
61	ND	1799.95	ND	14450.29	29262.10	49641.96	ND	ND	ND
62	2189.43	20697.98	ND	6965.75	22803.53	13431.12	ND	ND	ND
63	ND	9778.59	ND	2530.61	7839.58	3700.88	ND	ND	ND
64	ND	6188.82	ND	ND	1794.60	ND	ND	ND	ND
65	ND	87215.19	ND	29298.44	40173.29	6247.25	ND	ND	ND
66	4219.77	30505.13	ND	5771.66	12109.67	8649.01	ND	ND	ND
67	ND	2103.06	ND	19950.34	ND	ND	ND	ND	ND
68	2752.59	72049.91	ND	142068.56	156439.91	54841.88	ND	ND	ND
69	1143.17	404193.73	ND	236769.93	13467.65	15106.64	ND	ND	ND
70	ND	ND	ND	ND	14644.65	ND	ND	ND	ND
71	ND	6478.85	ND	1714.33	3865.00	ND	ND	ND	ND
72	ND	35942.25	ND	19764.80	30247.87	6376.75	ND	ND	ND
73	5966.76	113855.43	ND	28638.11	29648.77	75826.53	ND	ND	ND
74	6467.43	11942.60	ND	ND	ND	ND	ND	ND	ND
75	27165.36	152437.45	ND	78037.15	12586.65	1392.05	5759.60	ND	ND
76	7084.15	6187.25	ND	ND	ND	ND	ND	ND	ND
77	36021.36	76080.04	ND	32011.54	25074.93	41520.12	7499.54	ND	ND
78	15915.66	129718.56	ND	43788.92	112737.24	56303.96	3801.53	ND	ND
79	118853.81	818869.43	ND	333807.22	871883.28	397631.65	29712.99	ND	ND
80	2646.50	4176.48	ND	ND	ND	ND	ND	ND	ND
81	5100.72	11591.55	ND	11005.97	12926.32	1983.76	ND	ND	ND
82	ND	32800.47	ND	3773.28	ND	1770.73	ND	ND	ND
83	8113.54	40949.78	ND	13766.61	ND	ND	ND	ND	ND
84	45716.11	314291.23	ND	369919.70	246187.18	116090.27	11758.60	ND	ND

85	8609.28	41277.39	ND	25803.11	27724.85	19272.67	ND	ND	ND
86	8700.39	391608.25	ND	72172.26	36095.88	ND	ND	ND	ND
87	15935.92	28102.85	ND	7673.46	95873.44	98623.98	ND	ND	ND
88	3084.20	56921.88	ND	ND	20574.53	10055.08	ND	ND	ND
89	ND	ND	ND	108506.29	366215.51	34192.44	ND	ND	ND
90	16044.25	268972.05	ND	33458.69	27742.27	20464.92	ND	ND	ND
91	ND	8281.56	ND	ND	ND	4985.21	ND	ND	ND
92	6560.94	44341.02	ND	16563.90	ND	ND	ND	ND	ND
93	15671.57	48432.33	ND	4096.28	17796.50	15682.36	2749.40	ND	ND
94	2205.60	11538.85	ND	3173.73	ND	ND	ND	ND	ND
95	ND	1579.88	ND	10219.14	4571.93	ND	ND	ND	ND
96	ND	4728.01	ND	1352.28	4218.35	ND	ND	ND	ND
97	51892.39	1493282.89	ND	522061.40	473458.85	258619.65	8482.52	ND	ND
98	9152.30	89461.85	ND	29034.75	1854.74	ND	ND	ND	ND
99	12444.07	13344.52	ND	ND	ND	3475.05	ND	ND	ND
100	16466.56	37016.38	ND	17856.75	7535.48	25119.70	ND	ND	ND
101	13701.74	14236.06	ND	3579.61	3696.42	8601.61	ND	ND	ND
102	ND	14344.14	ND	5339.73	8188.67	4347.30	ND	ND	ND
103	37469.90	755457.92	ND	285159.28	192968.24	188886.92	7425.81	ND	ND
104	14973.69	39588.58	ND	9461.59	20138.06	16744.81	1240.38	ND	ND
105	11368.42	534998.11	ND	92829.61	79504.37	82015.40	3608.48	ND	ND
106	ND	28418.00	ND	ND	9495.36	2078.96	ND	ND	ND
107	ND	1617.21	ND	1192.53	2827.25	ND	ND	ND	ND
108	22918.51	328417.17	ND	176919.14	200188.37	63777.81	ND	ND	ND
109	64662.43	270222.91	ND	183482.92	158255.47	122494.31	20510.14	ND	ND
110	17556.97	627993.91	ND	84204.01	82089.09	75511.34	2216.18	ND	ND
111	ND	4882.04	ND	ND	13047.30	7265.10	ND	ND	ND
112	ND	32098.89	ND	ND	7102.96	3878.93	ND	ND	ND
113	ND	8507.01	ND	46403.07	21450.29	ND	ND	ND	ND

114	4239.76	146512.72	ND	115350.99	35145.26	25814.04	ND	ND	ND
115	1458.88	22632.77	ND	12669.56	29772.98	9460.95	ND	ND	ND
116	1766.48	33807.06	ND	16511.20	ND	14116.06	ND	ND	ND
117	ND	ND	ND	12143.46	ND	ND	ND	ND	ND
118	ND	6194.09	2769.14	ND	ND	ND	ND	ND	ND
119	4069.42	ND	8488.11	ND	ND	ND	ND	ND	ND
120	53788.12	6452.42	9039.54	ND	ND	ND	ND	ND	ND
121	1961.09	1459.22	3745.80	ND	ND	ND	ND	ND	ND
122	25225.84	8716.90	34509.81	1285.17	ND	ND	ND	ND	ND
123	6166.83	3871.31	1475.96	3372.77	ND	ND	ND	ND	ND
124	97783.28	3427.90	136125.99	ND	ND	ND	ND	ND	ND
125	ND	9682.04	23636.71	15622.94	ND	ND	ND	ND	ND
126	122238.57	26750.06	136580.79	4378.40	6781.62	ND	ND	ND	ND
127	21625.99	30707.05	83150.73	4212.01	ND	ND	ND	ND	ND
128	194635.18	27162.05	93989.75	ND	ND	ND	ND	ND	ND
129	90252.28	37537.68	15947.25	26779.59	14927.91	ND	ND	7959.44	ND
130	17263.68	149506.16	4095.66	15227.45	1816.83	ND	ND	ND	ND
131	ND	1295.72	1260.85	ND	ND	ND	ND	ND	ND
132	779958.30	242902.29	409680.44	114158.98	9198.53	10325.82	ND	ND	ND
133	3416.20	2793.43	ND	3194.68	ND	ND	ND	ND	ND
134	6031.34	279655.89	646352.24	25009.52	1556.71	ND	ND	ND	ND
135	88370.14	139945.06	198971.53	11084.94	ND	ND	ND	ND	ND
136	256344.58	29099.00	61100.06	4059.43	ND	4392.64	ND	ND	ND
137	231204.73	25035.77	85198.62	ND	3628.17	10599.62	8726.90	ND	13643.13
138	1606236.39	809201.91	997252.29	243316.46	16767.71	17813.99	ND	ND	2813.88
139	1386.67	ND	ND	ND	ND	ND	ND	ND	ND
140	62034.03	72101.36	67311.22	15225.84	ND	ND	ND	ND	ND
141	ND	ND	2067.78	ND	ND	ND	ND	ND	ND
142	29547.71	ND	ND	ND	1312.57	1673.54	ND	ND	ND
143	ND	68825.70	5705.54	2324.25	ND	ND	ND	ND	ND

144	7441.97	279347.67	470625.64	51871.42	2227.49	1366.67	ND	ND	ND
145	2796.68	ND	ND	ND	ND	ND	ND	ND	ND
146	63212.10	ND	ND	16207.43	19130.50	11747.47	8587.15	15350.41	19887.52
147	11125.89	8650.51	6534.18	10880.60	1160.23	ND	ND	ND	ND
148	ND	10445.88	ND	ND	9401.83	ND	ND	ND	ND

ND: non detected.

Table S4. Antioxidant properties of the tested extracts.

**Solvents**	**DPPH (mg TE/g)**	**ABTS (mg TE/g)**	**CUPRAC (mg TE/g)**	**FRAP (mg TE/g)**	**MCA (mg EDTAE/g)**	**PBD (mmol TE/g)**
Hexane	70.19±2.42^{e}	172.56±6.15^{f}	97.41±1.42^{e}	84.11±1.66^{f}	9.55±0.53^{e}	1.27±0.04^{e}
Ethyl acetate	938.84±0.55^{a}	4009.94±18.45^{a}	1377.38±49.12^{b}	1196.05±29.33^{c}	11.88±0.24^{d}	3.94±0.12^{a}
Dichloromethane	93.25±0.44^{e}	398.03±3.66^{e}	154.43±0.91^{e}	136.68±2.67^{f}	14.82±0.51^{c}	1.51±0.05d
Ethanol	935.68±1.08^{a}	4012.39±15.59^{a}	1332.52±21.37^{b}	1214.23±47.64^{c}	10.26±0.13^{e}	3.27±0.06^{bc}
Ethanol/water	939.35±1.78^{a}	4026.50±14.06^{a}	1730.38±89.51^{a}	1534.85±42.81^{a}	14.14±0.19^{c}	3.48±0.07^{b}
Water	933.74±3.22^{a}	3973.50±56.53^{a}	1278.06±62.55^{b}	1432.77±2.73^{b}	17.49±0.69^{b}	3.81±0.08^{a}
NADES-A	829.11±43.29^{b}	2830.66±67.42^{b}	833.38±6.17^{c}	968.98±19.43^{d}	21.76±0.72^{a}	3.48±0.04^{b}
NADES-B	767.55±17.15^{c}	2285.15±13.73^{c}	774.94±20.01^{cd}	860.90±6.08^{e}	na	3.32±0.12^{b}
NADES-C	712.48±28.19^{d}	2134.94±31.58^{c}	699.44±8.78^{d}	799.20±16.74^{e}	6.87±0.70^{f}	3.09±0.08^{c}

Values are reported as mean ±SD of three parallel measurements. TE: Trolox equivalent; EDTAE: EDTA equivalent; na: not active. Different letters in same column indicate significant differences in the tested extracts ($p<0.05$)

Table S5. Enzyme inhibitory of the tested extracts.

Solvents	AChE (mg GALAE/g)	BChE (mg GALAE/g)	Tyrosinase (mg KAE/g)	Amylase (mmol ACAE/g)	Glucosidase (mmol ACAE/g)
Hexane	7.32±0.14[e]	1.39±0.04[d]	49.14±0.34[f]	0.50±0.01[c]	1.00±0.12[bc]
Ethyl acetate	9.00±0.01[b]	1.73±0.06[bc]	81.29±0.12[d]	0.54±0.01[c]	0.93±0.02[c]
Dichloromethane	8.03±0.27[d]	1.52±0.06[cd]	60.27±0.78[e]	0.54±0.01[c]	1.01±0.05[bc]
Ethanol	9.00±0.02[b]	2.13±0.18[a]	78.41±0.64[d]	0.35±0.01[d]	1.01±0.01[bc]
Ethanol/water	8.88±0.09[bc]	1.87±0.09[b]	79.94±0.40[d]	0.40±0.02[d]	1.02±0.01[bc]
Water	8.63±0.02[c]	na	80.55±0.19[d]	1.01±0.03[b]	1.06±0.01[b]
NADES-A	15.16±0.20[a]	na	153.97±0.96[b]	0.38±0.01[d]	2.15±0.01[a]
NADES-B	na	na	94.25±2.59[c]	2.49±0.07[a]	2.17±0.01[a]
NADES-C	na	na	164.12±0.61[a]	2.54±0.04[a]	2.20±0.01[a]

Values are reported as mean ±SD of three parallel measurements. GALAE: Galantamine; KAE: Kojic acid; ACAE: Acarbose equivalent; na: not active. Different letters in same column indicate significant differences in the tested extracts ($p<0.05$)
